# Estradiol Reduces Ferrous Citrate Complex-Induced NOS2 Up-Regulation in Cerebral Endothelial Cells by Interfering the Nuclear Factor Kappa B Transactivation through an Estrogen Receptor β–Mediated Pathway

**DOI:** 10.1371/journal.pone.0084320

**Published:** 2013-12-23

**Authors:** Li-Ching Chen, Wen-Sen Lee

**Affiliations:** 1 Graduate Institute of Medical Sciences, College of Medicine, Taipei Medical University, Taipei, Taiwan; 2 Department of Physiology, College of Medicine, Taipei Medical University, Taipei, Taiwan; II Università di Napoli, Italy

## Abstract

Hemorrhagic stroke caused leakage of red blood cells which converts to hemoglobin, heme, and iron accumulated at the lesions. High concentration of ferrous iron from subarachnoid hemorrhage (SAH) induced cerebral vasospasm. Using the two-hemorrhage SAH model in rats, we previously demonstrated that estradiol (E2) significantly attenuated the SAH-induced vasospasm by inhibiting the NOS2 expression. Adding ferrous citrate (FC) complexes to the primary cultured mouse cerebral endothelial cells (CEC) to mimic the SAH conditions, we also showed that FC up-regulates NOS2 through nuclear translocation of NFκB induced by free radicals generation. Here, we further studied the molecular mechanism underlying E2-mediated reduction of the FC-induced up-regulation of NOS2. Treatment with E2 (100 nM) reduced the FC (100 µM)-induced increases of free radical generation and the levels of NOS2 mRNA and protein in the CEC. Moreover, E2 also prevented the FC-induced increases of IκBα phosphorylation, NFκB nuclear translocation, NFκB binding onto the NOS2 promoter, and the NOS2 promoter luciferase activity. However, knock-down the estrogen receptor β (ERβ), but not ERα, abolished the E2-mediated prevention on the FC-induced increases of NOS2 mRNA and protein. The data from the present study suggest that E2 inhibited NOS2 gene expression by interfering with NFκB nuclear translocation and NFκB binding onto the NOS2 through an ERβ-mediated pathway. Our results provide the molecular basis for designing the applicable therapeutic or preventive strategies in the treatment SAH patients.

## Introduction

Cerebral vasospasm is one of the major causes of mortality and morbidity in SAH patients [1]. The comprehensive critical care management for patients with SAH is crucial to optimize their recovery [2]. To date, however, the main therapeutic approaches remain elusive and the responses of treatment are inconsistent [3]. The pathogenesis of symptomatic vasospasm is complex and still can not be fully explained. Current studies indicate that iron in the ferrous state can cause vasospasm [4], [5]. In the physiological status, iron is bound and inactivated by transport proteins (e.g. transferrin) and intracellular storage proteins (e.g. ferritin) [6]. However, pathological circumstances can result in the presence of unbound iron in the brain. One of such circumstances is intracerebral hemorrhage, in which hemoglobin from red blood cells are cleaved to biliverdin by heme oxygenase in astrocytes and microglia, thereby releasing iron [7]. The iron released from heme is highly toxic to neurons [8]. In vivo studies have shown that hypoxic/ischemic conditions cause neuronal cell death and the affected area is accompanied by an increased level of iron and ferritin in microglial cells in cerebral cortex and hippocampus [9]–[11]. Intracerebroventricular injection of ferrous ammonium citrate induces the expression of toxic lipid peroxidation product, 4-hydroxynonenal (HNE), in CA3 of the hippocampus [12].

It has been shown that the production of nitric oxide (NO), one of the important endothelium-derived relaxing factors, is decreased within 10 min after SAH in experimental animal models [13] and in humans [14]. In normal circumstances, NO is released from endothelial cells and diffuses to the adjacent smooth muscle cells, where it activates the soluble guanylate cyclase, which in turn increases the production of cyclic guanosine monophosphate (cGMP), subsequently activates intracellular calcium pumps sequestering free Ca^2+^ into sarcoplasmic reticulum, and eventually causes relaxation of smooth muscle cells. Conversely, in the hemorrhagic conditions, NO is bound by oxyhemoglobin, bilirubin, or iron, subsequently causes a decrease of the guanylate cyclase activity, thereby reduces the cGMP production and causes vasospasm [2], [15]–[17]. NOS consist of different subtypes including neuronal (NOS1), inducible (NOS2), and endothelial (NOS3) enzyme [18]. NOS2 can be induced in a wide variety of cells, and its presence is associated with inflammation. NOS1 and NOS2 are harmful to ischemic brain and can induce neurotoxicity, while NOS3 is a protective enzyme with vasodilatory effects in the early stages of ischemia. Exploration of NOS2 expression suggests a link between the “inflammatory” form of NOS and vasospasm [19]–[24]. An immunohistochemical study of NOS2 expression demonstrated that a significant NOS2 immunoreactivity was observed in endothelial, muscular, and adventitial cells at 7 days post-SAH in the rat [25]. The increase of NO availability immediately after ischemia is beneficial because it can inhibit further decreases of cerebral blood flow and adhesion of platelets and leukocytes to micro-vessels [2].

Previous studies showed that men suffer from higher incident of stroke than premenopausal women [26], [27]. Estrogens have been suggested to control the cellular level of reactive oxygen species (ROS) and nitric oxide (NO) generation in normal healthy premenopausal women [28]. Long term estrogen treatment increases the protein levels of eNOS and enhances endothelial vasodilator function in cerebral arteries [28], [29]. Our previous in vivo study demonstrated that E2 treatment prevented the SAH-induced cerebral vasospasm in male rats through increasing the association of p65/ER and reducing the levels of NOS2 protein and mRNA [30]. However, the molecular mechanisms underlying E2-mediated protective effects are not well understood. Accordingly, we used the cultured primary mouse CEC to examine the molecular mechanism underlying E2-reduced the FC-induced increases of NOS2.

## Materials and Methods

### Chemicals

The FC complex was prepared as previously described [31]. Briefly, ferrous ammonium sulfate, citric acid, and estradiol were purchased from Sigma-Aldrich (St. Louis, MO, USA). ER antagonist, ICI 182780, was obtained from Tocris Bioscience (Ballwin, MO). Chemicals used in this study were dissolved in dimethyl sulfoxide (DMSO) or water according to the manufacturer's protocol.

### CEC culture

All animal protocols were approved by the Animal Care and Use Committee of the Taipei Medical University (licenses No. LAC-97-0160). All procedures were performed in compliance with the National Institutes of Health Guide for the care and use of Laboratory animals. The surgery was performed under isoflurane anesthesia to minimize suffering. The CEC was prepared as previously described [32]. Briefly, the Balb/c mouse was sacrificed by decapitation, meninges and white matter were removed, and cortices were minced and gently dissociated in Hank's balanced salt solution (GIBCO, Grand Island, NY). The resulting microvessel fraction was then sequentially digested with collagenase/dispase at a concentration of 1 mg/mL (Sigma-Aldrich) for 6 h at room temperature. After centrifugation, the pellet containing the CEC was washed with Dulbecco's modification of Eagle's medium (DMEM, GIBCO), maintained in DMEM supplemented with 10% fetal bovine serum (FBS) in a humidified incubator (37°C, 5% CO_2_). The CEC showed positive immunoreactivity for Von Willebrand factor (vWF), a marker for endothelial cells. Cells from passages 10 to 25 were used.

### Intracellular ROS level determination

For measurement of the intracellular ROS levels, cells were incubated with 5 µM 5- (and-6)-chloromethyl-2,7-dichlorodihydrofluorescein diacetate acetyl ester [CM-H2DCFDA (here referred to as DCF) (Invitrogen, Carlsbad, CA)] for 10 min, and then washed with phosphate-buffered saline (PBS). The fluorescence and differential interference contrast images were taken using Leica TCS SP5 fluorescent microscope imaging system (Wetzlar, Germany) and analyzed using flow cytometry according to the manufacturer's instructions (Becton Dickinson, San Jose, CA).

### Reverse transcriptase-polymerase chain reaction (RT-PCR) analysis

The RT-PCR assay for detecting the level of NOS2 mRNA was performed as described previously [33]. Briefly, the CEC was treated with the FC complex for 16 h, and then processed for total RNAs isolation using Trizol reagent according to manufacturer's protocol (Invitrogen). The cDNA was amplified from 1 µg of total RNA using a SuperScript one-step RT-PCR with platinum Taq system (Life Technologies, Karlsruhe, Germany). PCR was conducted for 35 cycles in thermal controller. Primers used for amplification were as follows: NOS2 forward primer: 5′-TGCTGTTCTCAGCCCAACAA-3′, and reverse primer: 5′-GAACTCAATGGCATGAGGCA-3′; ERα forward primer: 5′-TGCACCATTGACAAGAACCG-3′, and reverse primer: 5′-GTTCAGCA TCCAACAAGGCA-3′; ERβ forward primer; 5′-GAATGGTCAAGTGTGGATCCAGGAG-3′, and reverse primer: 5′-CTCCATCCAGCAGTTTCCAAGAGG-3′. The PCR primers for G3PDH were forward primer: 5′-GCATGGCCTTCCGTGTTCCTA-3′, and reverse primer: 5′-CCTTCAGTGGGCCCTCAGATG-3′. The amplification profile involved denaturation at 94°C for 1 min, primer annealing at 58°C for 30 sec, extension at 72°C for 1 min, and repeated for 35 cycles.

### Western Blot Analysis

Western blot analysis was performed as described previously [34]. Briefly, cell lysates were prepared, electrotransferred, immunoblotted with antibodies, and then visualized by incubating with the enhanced chemiluminescence system (Amersham, Buckinghamshire, England). Monoclonal mouse NFκB and IκBα antibodies, and polyclonal rabbit Poly ADP-ribose polymerase (PARP) and ER antibodies were purchased from Santa Cruz, (CA, USA). Monoclonal mouse NOS2 and phospho-IκBα (p-IκBα antibodies were purchased from BD Bioscience (Clontech) and Cell Signaling Technology (Beverly, USA), respectively. The level of G3PDH (Abcam) was detected and used as the control for equal protein loading. The intensity of each band was quantified by densitometry analysis using Image Pro Plus 4.5 software program (Media Cybernetics, Silver Spring, MD) and pixel densities were normalized to that of the loading control in Western blot analysis.

### Chromatin immunoprecipitation analysis (ChIP)

ChIP assays were performed as described previously [35]. The anti-NFκB and anti-ER antibodies (Santra Cruz) were used for immunoprecipitation reactions. Primers specific for the detection of transcription factor binding regions from -460 bp to -250 bp of the NOS2 gene were designed. The sense primer was 5′-GGATACACCACAGAGTGATG-3′, and the anti-sense primer was 5′-CATATCAGCTTCAGTCCAGC-3′.

### Real-time quantitative PCR assay

Total RNA was isolated from the CEC using Trizol (Invitrogen, Carlsbad, CA) according to the manufacturer's protocol. The NOS2 subunit-specific primers were synthesized as previously described [36]. A LightCycler thermocycler (Roche Molecular Biochemicals, Mannheim, Germany) was used for the real-time PCR. The NOS2 mRNA fluorescence intensity was measured and normalized with the level of G3PDH using the built-in Roche LightCycler Software Version 4.

### Cell transfection and dual luciferase reporter assay

For transient transfection, Lipofectamine™ 2000 transfection reagent (Invitrogen) was used according to the manufacturer's protocol. Briefly, Lipofectamine and plasmid DNA (3.5 µg of PGL3 NOS2 and 50 ng of pRL-TK) were added to each well containing cells and Opti-MEMR I Medium, and then incubated in a humidified incubator at 37°C for 4 h. The medium was replaced and the cell was then incubated for an additional 24 h. After incubation, the cell was treated with FC (100 µM) for 16 h, lysed in passive lysis buffer (Promega), and then mixed with luciferase assay substrate (Dual-Glo luciferase reporter system; Promega). The Firefly and Renilla luciferase activities were measured with a 96-well luminometer and analyzed by skanIt™ software 2.4.1. (Thermo Fisher Scientific, Rocford, IL).

### Immunocytochemistry and confocal microscope examination

The CEC was seeded on glass coverslips, treated with vehicle or FC (100 µM) for 4 h, washed three times with PBS, and then fixed with 4% formaldehyde in PBS for 15 min. Cells were placed in blocking solution (PBS containing 15% FBS, 2% bovine serum albumin, and 0.1% saponin) for 45 min at room temperature, and then incubated with anti-p65 Rhodamine-conjugated and anti-ER Fluorescein isothiocyanate (FITC)-conjugated monoclonal antibody at a 1:100 dilution for 1 h at room temperature in blocking solution. To visualize nuclei, DNA was stained with Hoechst (1 µg/mL in PBS and 0.1% BSA) for 5 min. After washing three times with PBS, cells were viewed under a laser confocal spectral microscope imaging system (Leica, TCS SP5, Bannockburn, IL, USA).

### Small interfering RNA (siRNA)

Expression of ERα and ERβ was knocked-down in CEC with at least three independent small interfering RNAs (siRNAs). The target sequences of ERα and ERβ mRNA were selected to suppress ERα and ERβ gene expression. Non-target sequences of each siRNA were used as controls [37]. After BLAST analysis to verify that there were no significant sequence homologies with other human genes, the selected sequences were inserted into *Bgl*II/*Hin*dIII-digested pSUPER vectors to generate the pSUPER-ER (ER-Si) and pSUPER-non-target (ER-NT). Three different anti-sense siRNAs targeted against different parts of the ERα sequence are listed below: Si-1: 5′- GATCCCCGAAGAATAGCCCTGCCTTGTTCAAGAGACAAGGCAGGGCTATTCTTCTTTTTA-3′; Si-2: 5′-GATCCCCGGAGACTCGCTACTGTGCCTTCAAGAGAGGCACAGTAGCGAGTCTCCTTTTTA-3′; Si-3: 5′-GATCCCCATGCAAGAACGTTGTGCCCT TCAAGAGAGGGCACAACGTTCTTGCATTTTTTA-3′ and NT: 5′- GATCCCCACGTGCGCATATGTACGCATTCAAGAGATGCGTACATATGCGCACGT-TTTTTA-3′. For ERβ siRNA sequences are: Si-1: 5′-GATCCCCTGACTATATCTGTCCAGCCTTCAAGAGAGGCTGGACAGATATAGTCATTTTTA-3′; Si-2: 5′-GATCCCCAGGTGTGGGTA CCGAATATTCAAGAGATATTCGGTACCCACACCTTTTTTA-3′; Si-3:GATCCCCGGTGTGGGTACCGAATAGTTCAAGAGACTATTCGGTACCCACACCTTTTTA and NT: 5′- GATCCCCGAGTGCCCCTCTCTATATATTCAAGAGATATATAGAGAGGGGCACTCTTTTTA-3′. All constructs were confirmed by DNA sequence analysis. The transfection protocol has been previously described [37], [38]. Briefly, 2×10^5^ cells were washed twice with PBS and mixed with 0.5 µg of plasmid, and then one electric pulse was applied for 10 ms under a fixed voltage of 1.4 kV on a pipette-type MP-100 microporator (Digital Bio, Seoul, Korea).

### Statistics

All data were expressed as the mean value ± s.e.mean. Comparisons were subjected to one way analysis of variance (ANOVA) followed by Fisher's least significant difference test. Significance was accepted at *P*<0.05.

## Results

### Effects of E2 on the FC-induced increases of intracellular ROS levels in the CEC

**Figure 1:**
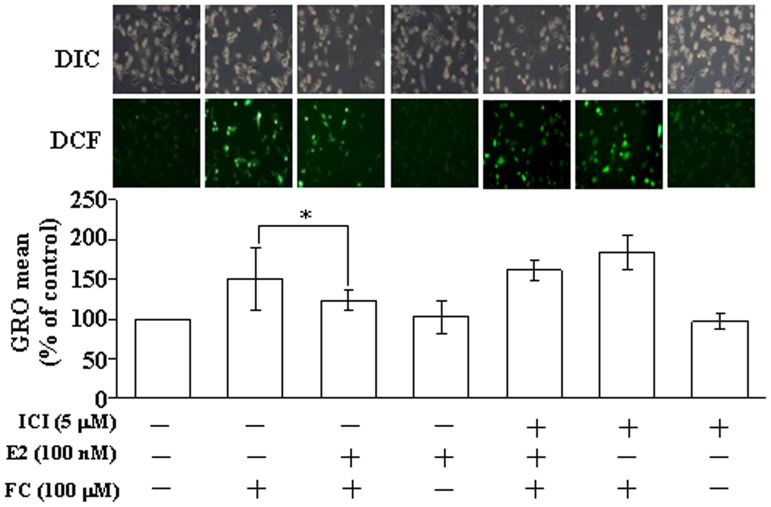
Effects of E2 on the FC-induced increases of intracellular ROS levels in the CEC. The FC-induced increases of ROS in the CEC were reduced by E2 (100 nM) treatment. This effect was blocked by pre-treatment of the cell with an ER antagonist, ICI (5 µM). Top panel: representative photographs of ROS generation. ROS levels were assayed using 5 µM DCF as described in Materials and Methods. DCF fluorescence images and DIC images were taken using Leica TCS SP5 fluorescent microscope imaging system (Wetzlar, Germany). Bottom panel: quantitative results of ROS levels. Values represent the means±s.e.mean. (n = 4). ^*^ *P*<0.05 different from FC-treated group. DCF, dichlorodihydrofluorescein diacetate [CM-H2DCF-DA]; DIC, differential interference contrast; E2, estradiol; FC, ferrous citrate; ICI, ICI 182,780.

Previously, we demonstrated that FC (1–100 µM) concentration-dependently increases the ROS level, which in turn activates phosphorylation of IκBα and nuclear translocation of NFκB, and results in activation of the NOS2 promoter and related protein transcription in the CEC [31]. In the present study, we further investigated the effect of E2 on FC-induced increases of the ROS level in the CEC. As shown in Figure 1, the level of ROS in CEC was increased at 5 min after FC (100 µM) treatment. Pre-treatment E2 (100 nM) for 1 h followed by co-treatment with FC (100 µM) and E2 (100 nM) together reduced the FC-induced increases of ROS. However, pre-treatment of the cell with an ER antagonist, ICI 182,780 (5 µM), abolished the E2-mediated reduction of the FC-induced increases of ROS.

### Effects of E2 on the FC-induced increases of the levels of NOS2 mRNA and protein in the CEC

**Figure 2:**
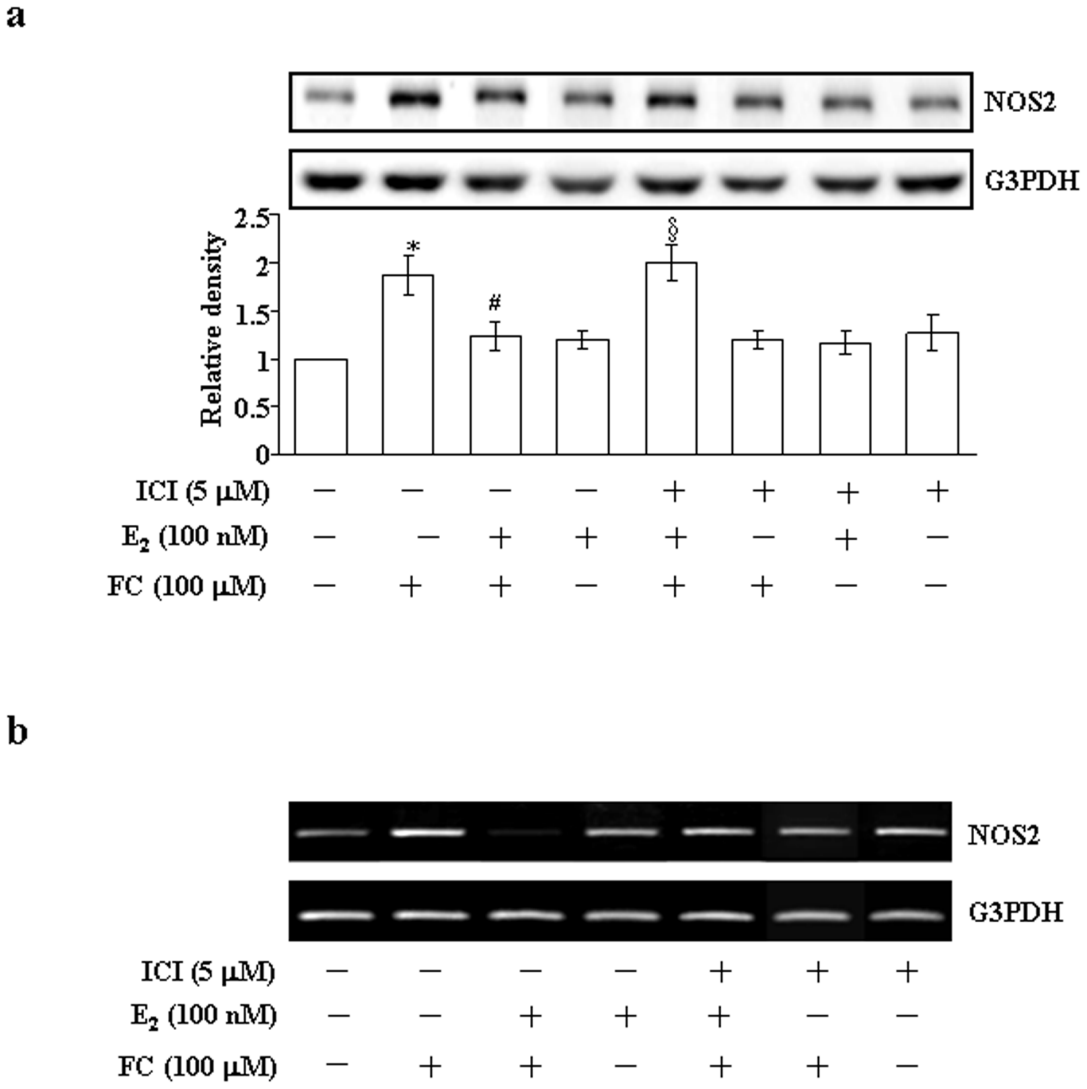
Effects of E2 on the FC-induced increases of the levels of NOS2 mRNA and protein in the CEC. The FC-induced increases of the levels of NOS2 protein (a) at 24 h after FC treatment and mRNA (b) at 16 h after FC (100 µM) treatment in the CEC were abolished by E2 (100 nM) treatment. This effect was blocked by pre-treatment of the cell with an ER antagonist, ICI (5 µM). Quantitative results of NOS2 protein levels, which were adjusted with G3PDH protein level and expressed as fold-induction of its own control. Values represent the means±s.e.mean. (n = 4). ^*^ *P*<0.05 different from control group. ^#^ *P*<0.05 different from FC-treated group. ^§^ *P*<0.05 different from combined treatment with FC and E2 group. E2, estradiol; FC, ferrous citrate; ICI, ICI 182,780.

We next examined the effect of E2 on the FC-induced up-regulation of NOS2. As illustrated in Figure 2, treatment of the CEC with FC (100 µM) increased the levels of NOS2 protein (Fig. 2a) and mRNA (Fig. 2b). These effects were abolished by E2 (100 nM) treatment, and pre-treatment with ICI 182,780 (5 µM) abolished the E2-mediated reduction of the FC-induced up-regulation of NOS2.

### Effects of E2 on the FC-induced IκBα phosphorylation and nuclear translocation of NFκB in the CEC

**Figure 3:**
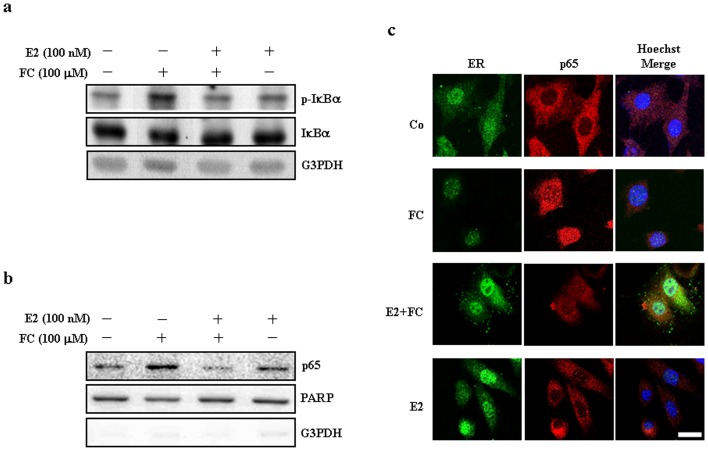
Effects of E2 on the FC-induced IκBα phosphorylation and nuclear translocation of NFκB in the CEC. (a) The FC-induced increases of the levels of p-IκBα in the CEC were abolished by E2 (100 nM) treatment. Membrane was probed with anti-G3PDH antibody to verify equivalent loading. (b) The FC-induced an increase of p65 nuclear translocation in the CEC was abolished by E2 (100 nM) treatment. PARP and G3PDH were used as a nuclear and cytosolic protein marker, respectively, to confirm the purities of isolation and to verify equivalent loading. (c) E2 and FC induced nuclear translocation of ER and p65, respectively. After treatment with FC for 4 h, the CEC was fixed and then labeled with an anti-p65 antibody followed by a Rhodamine-conjugated secondary antibody and an anti-ER antibody followed by a FITC-conjugated secondary antibody. The nuclei were visualized with Hoechst (1 µg/mL) staining as described in Materials and Methods. Bar  = 25 µm. Co, control; E2, estradiol; FC, ferrous citrate.

NFκB is a key transcription factor that mediates expression of NOS2 [39]. Previously, we demonstrated that FC increases IκBα phosphorylation and NFκB (p65) nuclear translocation in the CEC. In the present study, Western blot analyses demonstrated that E2 reduced the FC-induced increases of IκBα phosphorylation (Fig. 3a), and p65 nuclear translocation (Fig. 3b) in the CEC. The E2-induced reduction of FC-activated p65 nuclear translocation was confirmed by confocal microscopy (Fig. 3c).

### Effects of E2 on the FC-induced increases of p65 binding onto the NOS2 promoter and the NOS2 luciferase promoter activity in the CEC

**Figure 4:**
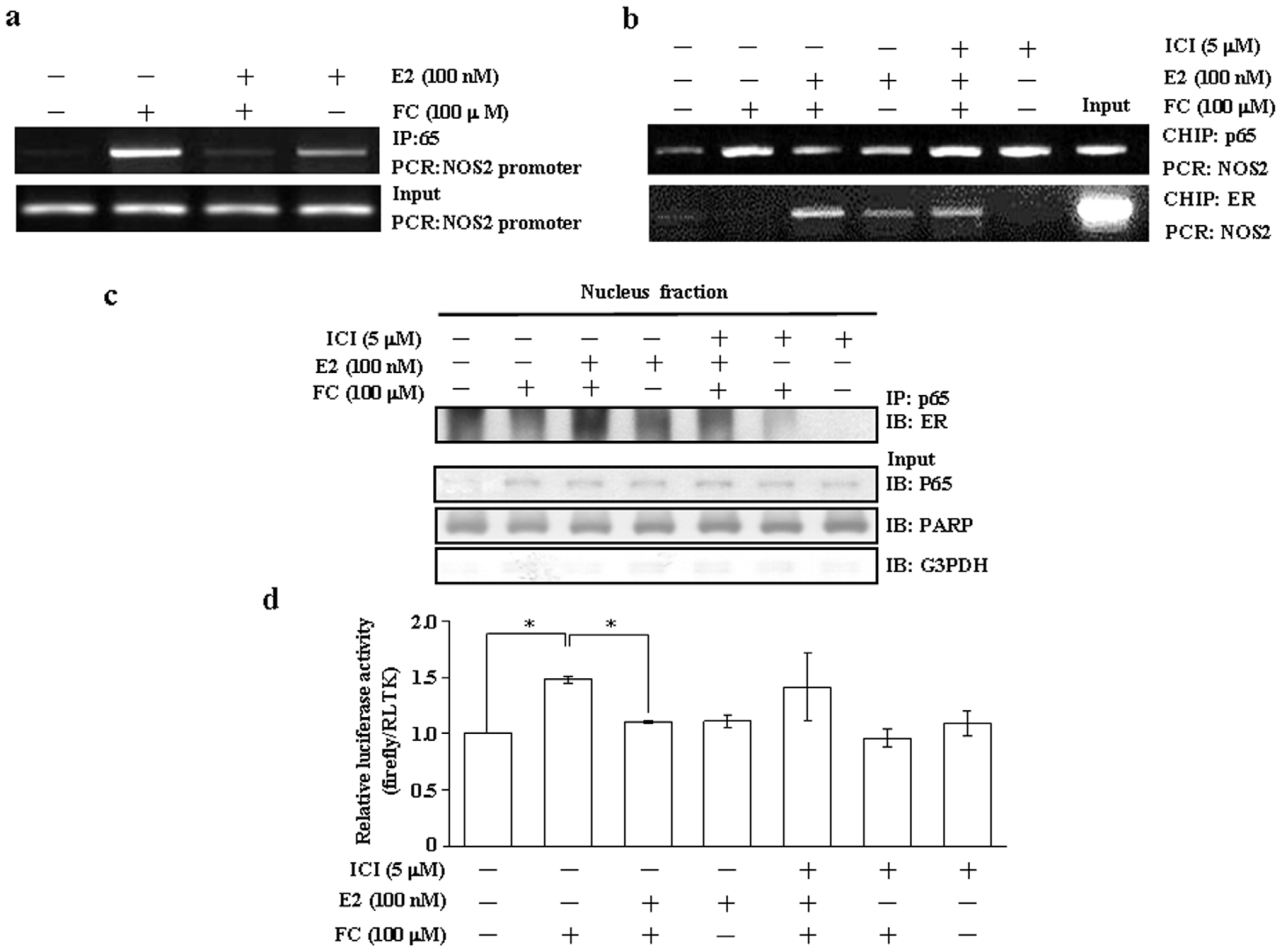
Effects of E2 on the FC-induced increases of NFκB binding onto the NOS2 promoter and NOS2 luciferase promoter activity in the CEC. (a) The FC-induced increases of p65 binding onto the NOS2 promoter DNA in the CEC was abolished by E2 (100 nM) treatment. (b) The prevention effect of E2 (100 nM) on the FC-induced increases of p65 binding onto the NOS2 promoter DNA in the CEC was blocked by pre-treatment of the cell with an ER antagonist, ICI 182,780 (5 µM). (c) E2 increased the formation of the p65-ER complex in the nucleus of the CEC. This effect was blocked by pre-treatment of the cell with ICI (5 µM). P65 was immunoprecipitated by anti-p65 antibody, and ER-p65 association was detected by anti-ER antibody. PARP and G3PDH were used as a nuclear and cytosolic protein marker, respectively, to confirm the purities of isolation and to verify equivalent loading. (d) The CEC was transiently transfected with the mouse NOS2 promoter DNA for 24 h, and then treated with vehicle, FC (100 µM), E2 (100 nM), ICI (5 µM), or in combination for 16 h. Subsequently, the cell was processed for the luciferase activity assay. Quantitative results of the NOS2 promoter activity were shown and expressed as fold induction of the CEC treated with vehicle (control). Values represent the means±s.e.mean. (n = 4). ^*^ *P*<0.05, different from cells treated with FC. CHIP, chromatin immunoprecipitation; IB, immunoblotting; IP, immunoprecipitation; E2, estradiol; ER, estrogen receptor; FC, ferrous citrate; ICI, ICI 182,780.

We next examined whether the binding of NFκB onto the NOS2 promoter was affected by E2 treatment. The ChIP assay demonstrated that E2 abolished the FC-induced increases of p65 binding onto the NOS2 promoter (Fig. 4a) and this effect was blocked by pre-treatment with ICI 182,780 (Fig. 4b). In the absence of E2, binding of ER onto the NOS2 promoter was not observed (Fig. 4b) and formation of the ER-p65 complex in the nucleus was rarely detected (Fig. 4c). In the presence of E2, however, formation of the ER-p65 complex (Fig. 4c) and binding of ER onto the NOS2 promoter (Fig. 4b) were increased, but binding of p65 onto the NOS2 promoter was decreased (Fig. 4b). The FC-induced increases of the NOS2 luciferase promoter activity were also abolished by E2 treatment (Fig. 4d). The E2-mediated increases of ER-p65 association and reductions of the NOS2 luciferase promoter activity were blocked by pre-treatment of the cell with ICI 182,780.

### Involvement of the ER subtype on the E2-mediated inhibition of the FC-induced NOS2 up-regulation

**Figure 5:**
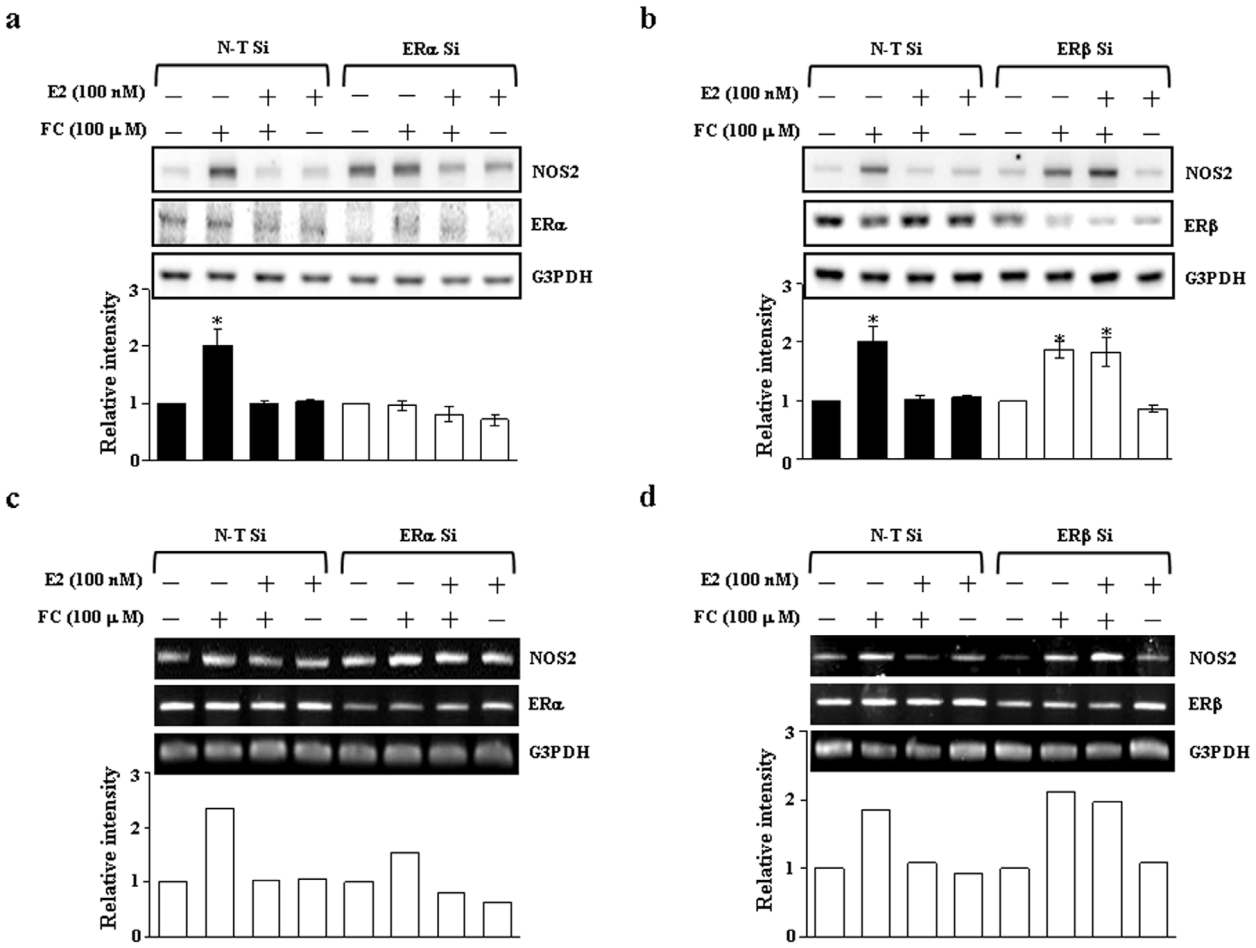
Involvement of the ER subtype on the E2-mediated inhibition of the FC-induced NOS2 up-regulation. (a) Pre-transfection of CEC with ERα siRNA did not significantly affect the E2-mediated inhibition on the FC-induced increases of the levels of NOS2 protein. (b) Pre-transfection of CEC with ERβ siRNA abolished the E2-mediated inhibition on the FC-induced increases of the levels of NOS2 protein. Top panel: representative results of NOS2 and G3PDH protein levels determined by Western blot analysis. Membrane was probed with anti-G3PDH antibody to verify equivalent loading. Bottom panel: quantitative results of NOS2 protein levels, which were adjusted with G3PDH protein level and expressed as fold-induction of its own control. Values represent the means±s.e.mean. (n = 3). ^*^ *P*<0.05, different from cells treated with FC. (c) Pre-transfection of CEC with ERα siRNA did not significantly affect the E2-mediated inhibition on the FC-induced increases of the levels of NOS2 mRNA. (d) Pre-transfection of CEC with ERβ siRNA abolished the E2-mediated inhibition on the FC-induced increases of the levels of NOS2 mRNA. The levels of mRNA were detected by RT-PCR (top panel) or real-time quantitative PCR (bottom panel). ER, estrogen receptor; N-T Si, non-target siRNA; Si, small interfering RNA.

We further examined which ER subtype is involved in the E2-mediated inhibition of the FC-induced NOS2 up-regulation. Pre-transfection of the CEC with ERα siRNA did not significantly affect the E2-mediated inhibition of FC-induced increases of the levels of NOS2 protein (Fig. 5a) and mRNA (Fig. 5c). In contrast, knock-down of ERβ abolished the E2-mediated inhibition of FC-induced increases of the levels of NOS2 protein (Fig. 5b) and mRNA (Fig. 5d).

## Discussion

Accumulating evidence has indicated that iron in the ferrous state could be a risk factor for the cerebral vasospasm [40], [41]. It has been demonstrated that intracerebral injection of lysed blood, hemoglobin, and FC could cause neurodegeneration due to redox cycling of iron complex, increases of hydroxyl radical, lipid peroxidation, oxidative stress, and brain injury [42]. Moreover, continuous intravenous administration of 2,2′-dipyridyl, a ferrous iron chelator, prevents delayed vasospasm in a primate model of SAH [4], [5]. Previously, we applied FC complexes to the primary cultured mouse CEC to mimic the SAH conditions and demonstrated that FC induced increases of the intracellular levels of ROS and NOS2, which has been suggested to be a critical factor for inducing cerebral vasospasm [31]. Moreover, injection of FC into the caudate nucleus caused brain injury in rats [43]. Our previous in vivo studies also showed that E2 prevented the SAH-induced vasospasm and increases of the levels of NOS2 protein and mRNA in basilar artery through an ER-dependent mechanism. We herein used the cultured mouse CEC to examine the effect of E2 on the induction of NOS2 by FC. The results of the present study demonstrate that E2 can reduce the FC-induced increases of ROS, and NOS2 mRNA and protein in the CEC.

It has been indicated that increased formation of ROS on re-perfusion after ischemia underlies ischemia re-perfusion damage [44]. ROS can directly or indirectly damage all biomolecules, including proteins, lipids, DNA, and carbohydrates [45]. Free oxygen radicals produced by hemoglobin, bilirubin or iron can damage cerebral endothelial and smooth muscle cells [46]. It can increase the endothelial permeability, intracellular calcium, and inositol 1,4,5-triphosphate levels, thereby cause cell depolarization. On the other hand, NO and peroxynitrite, a powerful oxidant formed by reaction of NO with superoxide ions, are neurotoxic. The mechanisms underlying the ROS-induced neurotoxicity may involve DNA damage and activation of poly adenosine diphosphate (ADP)-ribose synthase with subsequent depletion of glycerol-3-phosphate dehydrogenase and ATP or mitochondrial damage leading to necrosis and apoptosis. Previously, we demonstrated that FC can increase ROS generation and NOS2 gene expression in mouse CEC [31]. Our data also suggest that increases of ROS might contribute to the FC-induced up-regulation of NOS2 through activation and binding of NFκB onto the NOS2 promoter of the mouse CEC. In the present study, our data demonstrated that E2 could reduce the FC-induced increases of ROS generation (Fig. 1) through an ER-mediated pathway. At this point, we still do not know how the E2 down-regulates ROS level. It has been shown that ER is presented in the mitochondria, which represent the major source of ROS in the cell, and treatment with E2 could increase cytochrome c in the mitochondrial fraction [47]. Moreover, increases in cytochrome c have been demonstrated to significantly reduce complex III ROS [48], [49]. It has also been suggested that cytochrome c can directly supply electrons to superoxide, converting them to H_2_O and O_2_, thus reducing mitochondrial ROS. To study how the E2 down-regulates ROS level in the CEC is still ongoing.

Previously, it was demonstrated that NFκB is activated in the arterial wall after experimental SAH in rabbits [50]. This finding led the scientists to propose that activation of NFκB potentially leads to vasospasm development through induction of inflammatory response. It has been shown that NOS2 expression can be transcriptionally up-regulated by NFκB activation [51]–[53]. The 5′region of the *NOS2* gene contains NFκB-responsive element [30], suggesting that NOS2 is one of the NFκB-regulated genes. Our previous study confirmed that the two predicted NFκB binding sites exist in the NOS2 promoter within the range of -1529 bp to -1516 bp and -1224 bp to -1210 bp, and both NFκB binding sites are involved in the FC-activated NOS2 transcriptional activity [31]. It has been shown that intra-cisternal injection of decoy NFκB oligonucleotides could relieve the SAH-induced vasospasm in rabbits [54]. Under non-stimulating conditions, NFκB remains in the cytoplasm by tightly binding to the inhibitory IκBα protein [55]. Activation of NFκB is triggered by phosphorylation, ubiquitination, and degradation of the IκBα protein, which in turn results in dissociation of NFκB from IκBα, and thereby allows NFκB to migrate into the nucleus and activates gene expression [56], [57]. In this study, our data showed that E2 reduced the FC-induced increases of NFκB (p65) nuclear translocation through suppressing the phosphorylation of IκBα protein (Fig. 3). Although the molecular mechanism underlying E2-induced suppression of the FC-induced phosphorylation of IκBα protein was not investigated in the present study, it has been demonstrated that treatment of human coronary artery endothelial cells with E2 activated PI3K/Akt, p38, and JNK, all of which activated ERK1/2 followed by NFκB activation [58]. Moreover, our previous study demonstrated that pre-treatment of CEC with N-Acetyl-Cysteine (NAC), a reactive oxygen species (ROS) scavenger, abolished the FC-induced increases of NFκB nuclear translocation as well as NFκB binding onto the NOS promoter, and the levels of NOS2 mRNA and protein [31], suggesting that the changing of ROS level is one of the important factors that affect NOS2 induction. The effect of ROS on the activation of ERK and p38 has been demonstrated [59]. A detailed molecular mechanism underlying E2-mediated reduction of the FC-induced p65 nuclear translocation still deserves further investigation.

In the absence of E2, FC increased binding of p65 onto the NOS2 promoter (Fig. 4a). In contrast, E2 increased nuclear translocation of ER (Fig. 3c), binding of ER onto the NOS2 promoter (Fig. 4b), and formation of p65-ER complex (Fig. 4c). Using JASPAR computer analysis, it has been indicated that there are 4 potential ER binding sites (-1434 bp to -1445 bp, -1259 bp to -1269,-718 bp to -729 bp, -489 bp to -499 bp) on the NOS2 DNA promoter region. The functional interaction between ER and p65 has been previously demonstrated both *in vitro* and *in vivo* [60]. The potential for a reciprocal transcription inhibition between agonist-bound ER and activated p65 has been documented [61]. Several mechanisms including DNA binding have been suggested to be involved in this cross-talk [62]. Previously, our in vivo study demonstrated that E2 increases the association of p65/ER and hence reduces the binding between p65 and *iNOS* DNA [30]. Consistent with our in vivo study, the results from the present in vitro study showed that E2 increased the association of p65/ER in the nucleus and hence diminished the binding of p65 onto the NOS2 promoter through an ER-dependent pathway (Fig. 4b). Moreover, ER knock-down technique further suggested that ERβ, but not ERα, was involved in the E2-mediated inhibition of the FC-induced up-regulation of NOS2 (Fig. 5). Surprisingly, transfection with ERα siRNA increased the basal levels of NOS2 protein (Fig. 5a) and mRNA (Fig. 5c) in the CEC. These phenomena were not observed in the CEC transfected with ERβ siRNA or non-target siRNA (Figs. 5b and 5d). While we did not have a reasonable explanation for these phenomena, the E2-mediated inhibition of the FC-induced NOS2 up-regulation was observed in the CEC transfected with ERβ siRNA, but not ERα siRNA.

**Figure 6:**
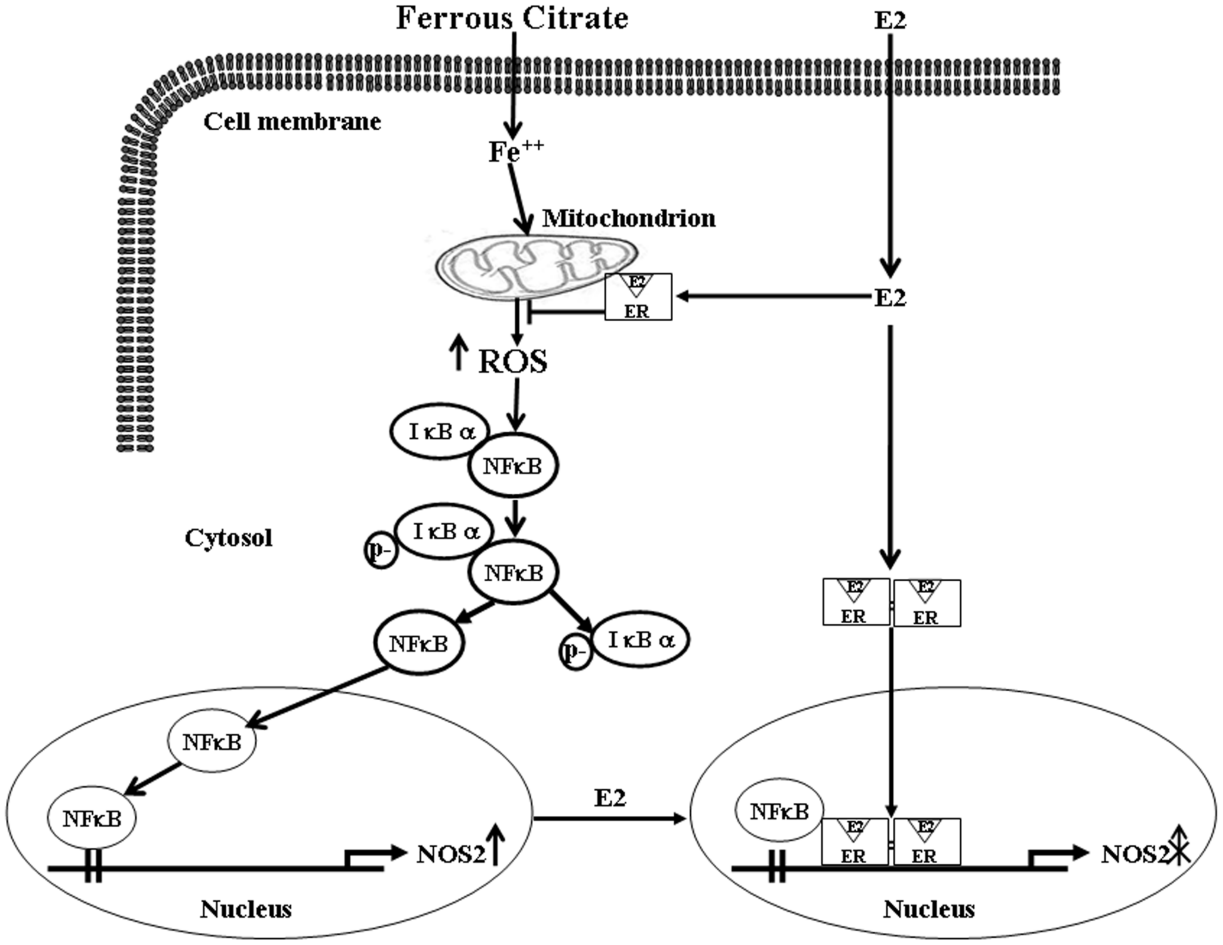
Proposed signaling pathway associated with E2-caused reduction of the FC-induced NOS2 up-regulation in the CEC. E2 suppressed the NFκB-induced increases of NOS2 through (1) maintenance of steady level of inhibitor IκBα in the cytoplasm; (2) prevention of cytosolic NFκB from translocation into the nucleus; and (3) interference of NFκB binding onto the *NOS2* promoter DNA by physical association of ERβ with NFκB.

In summary, the results of the present in vitro study suggest that E2 suppressed the NFκB-induced increases of NOS2 through (1) maintenance of steady level of inhibitor IκBα in the cytoplasm; (2) prevention of cytosolic NFκB from translocation into the nucleus; and (3) interference of NFκB binding onto the *NOS2* promoter DNA by physical association of ERβ with NFκB. These in vitro findings further support the conclusion from our previous in vivo studies showing that E2 might inhibit the SAH-induced increase of NOS2 by increasing the association of p65-ER, which in turn inhibits the binding of p65 onto the *NOS2* promoter DNA [30]. Based on the results from the present study and our previous study, we propose a model of the molecular mechanism underlying E2-mediated reduction of the FC-induced NOS2 up-regulation in CEC (Fig. 6). The findings from our previous in vivo and the present in vitro studies suggest the potential applications of E2 in treatment of SAH patients.
